# The Growing Genetic and Functional Diversity of Extended Spectrum Beta-Lactamases

**DOI:** 10.1155/2018/9519718

**Published:** 2018-03-26

**Authors:** Sadeeq ur Rahman, Tariq Ali, Ijaz Ali, Nazir Ahmad Khan, Bo Han, Jian Gao

**Affiliations:** ^1^Department of Clinical Veterinary Medicine, College of Veterinary Medicine, China Agricultural University, Beijing, China; ^2^Abdul Wali Khan University, Garden Campus, Khyber Pakhtunkhwa, Pakistan; ^3^Department of Biosciences, COMSATS Institute of Information Technology (CIIT), Bioscience Block, Chak Shahzad Campus, Park Road, Islamabad, Pakistan; ^4^Department of Animal Nutrition, The University of Agriculture, Peshawar, Pakistan

## Abstract

The *β*-lactams—a large class of diverse compounds—due to their excellent safety profile and broad antimicrobial spectrum are considered to be the most widely used therapeutic class of antibacterials prescribed in human and veterinary clinical practices. This, unfortunately, has also given rise to a continuous increased resistance globally in health care settings as well as in the community due to their permanent selective force driving diversification of the resistance mechanism. Resistance against *β*-lactams is increasing rapidly as novel *β*-lactamases, enzymes that degrade *β*-lactams, are being discovered each day such as recent emergence of extended spectrum *β*-lactamases (ESBL) that have the ability to inactivate most of the cephalosporins. The complexity and diversity of ESBL are increasing so rapidly that more than 170 variants have thus far been described for only a single genotype, the *bla*_CTX-*M*_-encoding ESBL. This review is to organize all the current updated literature describing genomic features, organization, and mechanism of resistance and mode of dissemination of all known ESBLs.

## 1. Antibiotics and Resistance

Antibiotics or antimicrobials are antibacterial drugs, which refer to natural metabolites of microorganisms such as fungi, actinomycetes, and bacteria and are used for killing or restricting growth of target microorganisms. Generally, it is perceived that antibiotic production is triggered to compete for available space and nutrients and is likely vital for the survival and persistence of the host organisms. Enzymes that inactivate other antibiotics and develop resistance against them are secreted by the host bacterium as like other secretory products of bacterial cells such as TpsA proteins, which are also thought to secrete to kill other competing similar bacteria [1–4]. Sir Alexander Fleming in 1928 observed that an opportunistic fungus,* Penicillium notatum*, was able to deny the growth of* S. aureus* on an agar plate and in fact that novel discovery led to the road of application of those antimicrobial compounds against the evil microorganisms. The secreted inhibitory substance “penicillin” was thus soon showcased in the market that saved many lives during 1941 in England [5]. The discovery of penicillin and its potential use for restricting many infections inspired many scientists to look for other natural compounds and the quest of that golden antibiotic discovery era from 1945 to 1980 led to the introduction of many successful antibiotics currently used in clinics. Remarkably, during this era, not only that numerous of new categories of antimicrobials were discovered, but they were also made available in the market for use against infections. From 1980s onward until recently 2000s, alongside discovering new antibiotics, emphasis was also made on improvements of already available antimicrobial drugs. Although discovery of the new antibiotics remained low during the last few years, yet, mining for more efficient and safe drug is still an ongoing and unending expedition. Drug designing and finding new drug targets remained a hot issue in the last decades in the field of medicines and recently a lot has been invested in formulating and designing new synthetic drugs that could replace antibiotics. Currently, in the shelf of antibiotics, there are drugs that are either semisynthetic, a modified natural product, or synthetic that is chemically designed in the laboratories, such as sulphonamides and quinolones [6]. Based on the diversity of the origin of these compounds, there are generally three different bases of antibacterial classification: (i) chemical structure, (ii) target site of the drugs, and (iii) impact of final outcome on the target such as bactericidal or bacteriostatic nature. Categorization based on the end effect (bactericidal or bacteriostatic) can be ambiguous, because some drugs have bactericidal effect against one bacterium and bacteriostatic effect against another. Categorization based on the chemical structure is inadequate because of the diversity among agents. Finally, grouping on the basis of target site is more appropriate because it helps in understanding the molecular basis and mode of action of antibacterial action. In conclusion, antibiotics usually exert its effects through one of the five mechanisms: (i) cell wall synthesis inhibition (penicillins, monobactams, carbapenems, and bacitracin), (ii) cytoplasmic membrane inhibition (polymyxins), (iii) bacterial protein synthesis inhibition (chloramphenicol, lincosamides, macrolides, aminoglycosides, tetracycline, etc.), (iv) nucleic acid synthesis blocker (quinolones, nitroimidazoles, and rifampicin), and (v) folic acid synthesis blocker (sulphonamides, trimethoprim, etc.) [7].

The *β*-lactam drugs are the most important and frequently used groups of antimicrobials that inhibit cell wall synthesis resulting in lysis of specifically bacterial cells and thus are bactericidal. They have been categorized, on the basis of the chemical structure with the *β*-lactam ring, into six major groups: penicillins, cephalosporins, cephamycins, carbapenems, monobactams, and *β*-lactamase inhibitors [8]. With the introduction of penicillin for the first time, people were optimistic to end the dominance of bugs and overcome the evils of pathogens; however, very soon, penicillin was seen ineffective against some infections due to emergence of resistance against penicillin and the optimism was slashed [9]. Nevertheless, this phenomenon of emergence of resistance had accelerated the quest of human to overcome the resistance phenomenon by discovering next-generation antibiotics such as cephalosporin. The quest of discovering new antibiotics has begun in order to discover drugs that can kill all existing pathogens which led to the discovery of 2nd-generation (cefoxitin, cefotetan, cefmetazole, cefaclor, cefpodoxime, and cefuroxime), 3rd-generation (cefixime, cefodizime, cefotaxime, cefteram, and ceftizoxime), and 4th-generation (cefepime, cefluprenam, cefoselis, cefozopran, cefpirome, and cefquinome) cephalosporin drugs; however, at the same time bugs are fighting back for dominance and looking for new traits to adopt (Figure 2). Meanwhile, the advancement of technology to unravel genetic composition in parallel with improvements in transcriptomic analysis helped biotech industry to engineer genetically modified strain with better production of improved antibiotics, such as engineering of* Streptomyces hygroscopicus* 5008 through metabolic engineering for enhanced production of validamycin [10, 11]. Such an improvement towards production of new natural secreted antibiotics in response to enzymes that inactivate beta-lactams and carbapenems would certainly help overcoming shortage of new antibiotics. No doubt, there would have been a pool of resistance elements against all those naturally effective drugs and smart bugs were able to finally acquire them from nature and display them for their own defence translating emergence of horrible broad resistance against those otherwise highly effective recently discovered drugs (Figure 2).

Antibiotics of *β*-lactam group are used globally and approximately 50% of all prescribed antimicrobial belong to this group; however, unfortunately, resistance to this important safe and efficient class of antibiotic is increasing worldwide [12, 13]. Emergence and increasing occurrence of resistance is putting a lot of pressure and presents challenge to healthcare experts. Resistance to *β*-lactam compounds is mainly due to the production of beta-lactamases (BLs) that hydrolyze and thereby inactivate beta-lactam antibiotics [14]. The complexity and heterogeneity of BLs can be estimated from the constantly increased discoveries, and today more than 900 types, produced by many different species of bacteria, are recognized. Because of the broad spectrum activity against the latest cephalosporin (the extended spectrum cephalosporins), the extended spectrum beta-lactamases (ESBLs) are of specific concern. Blind and persistent use of antibiotics is thought to be one of the important triggers to provoke and spread antibiotic resistance. The objectives of the current manuscript is to accumulate concise updates regarding resistance offered by ESBL producing* Enterobacteriaceae* with special emphasis on structural and functional diversity, clinical significance, and global epidemiology.

## 2. The **β**-Lactam Antibiotics

**Figure 1:**
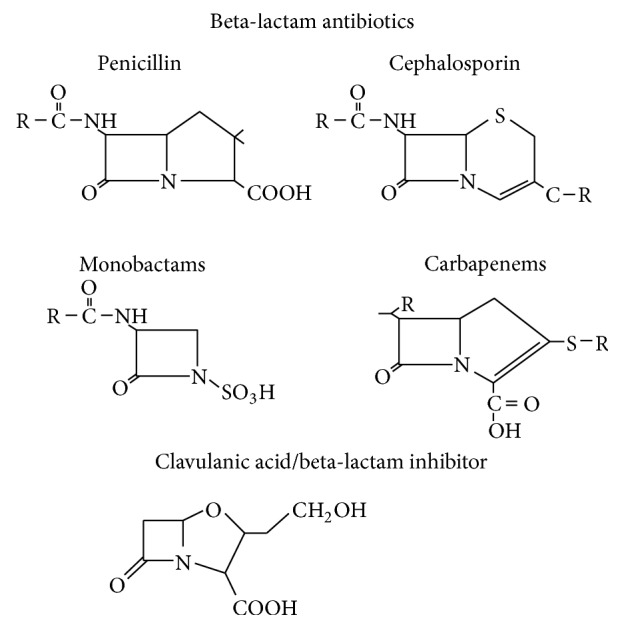
Structure of *β*-lactam compounds.

**Figure 2:**
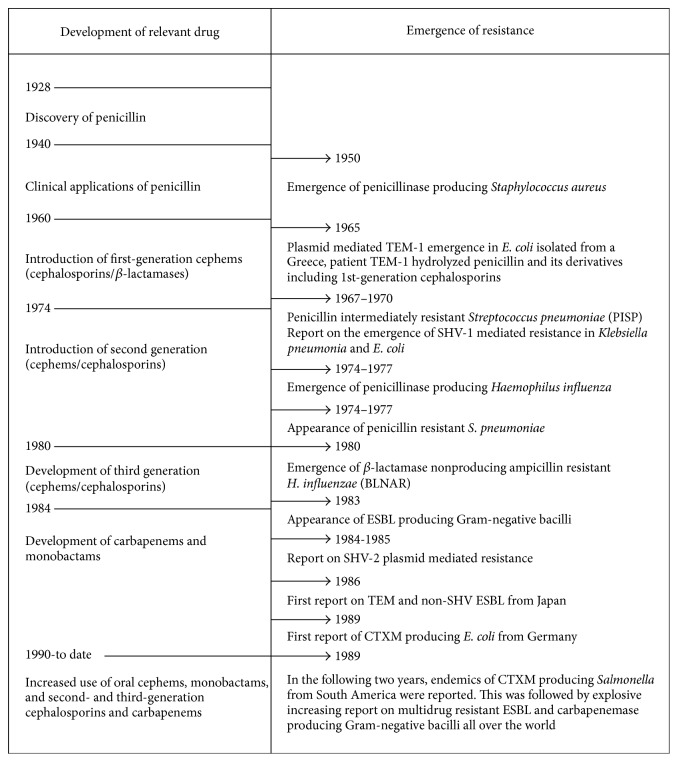
Trend of development of antibiotics and emergence of resistance with particular emphasis on ESBL.

The *β*-lactams are a group of antibacterial comprising four major groups: penicillins, cephalosporins, monobactams, and carbapenems [7]. Structurally, they consisted of a *β*-lactam ring, which is consisting of three carbon atoms and one nitrogen atom and is linked to a thiazolidine ring. In cephalosporins, the *β*-lactam ring and dihydrothiazine ring are merged; however, in the carbapenems, the *β*-lactam ring is joined with a hydroxyethyl side chain, deficient of an oxygen or sulphur atom in the bicyclic nucleus, while monobactam has no additional ring (Figure 1) [7].

### 2.1. Mechanism of Action of *β*-Lactams

There has been enormous improvement in understanding physiological principles of drug action, its metabolites, and fate by using the latest state-of-the-art genomic and functional techniques. No doubt, on-hand current improved technology helped us to elucidate many unknown principles and mechanism of actions and emergence of resistance of many antibacterial drugs. The *β*-lactams execute their antibacterial activity by inhibiting bacterial cell wall, peptidoglycan, and synthesis by preventing precise functioning of penicillin binding protein, which is also known as transpeptidases [50]. Peptidoglycan is a crucial structural constituent of the bacterial cell and periplasmic part. Apart from rigidity, it provides protection from the high internal osmotic pressure and gives an overall defined shape to a bacterial cell. PBP catalyzes cross-linking between amino acids of adjacent chains of amino acids that build into a mesh in the periplasmic space between the inner and outer membrane. Interestingly, *β*-lactam ring is similar to that of D-alanine-D-alanine of the N-acetylmuramic acid (NAM) pentapeptide; and thus PBPs “mistakenly” (due to very close shape resemblances) pick these up (*β*-lactam in fact) to use them as building blocks during cell wall synthesis. Bacterial cell pays for this mistake that leads to acylation of the PBP and thus eventually renders the enzyme (transpeptidases) inactive with inhibition of the transpeptidation reactions resulting in accumulation of cell wall precursor units that trigger activation of the cell wall autocatalytic system, leading to cell lysis [51]. By simultaneously blocking transpeptidases and activating autolysin, *β*-lactams lead to disruption of the synthesis of cell wall and initiates its active destruction finally resulting into cell lysis and elimination of the bug.

### 2.2. Mechanism of Bacterial Resistance to *β*-Lactam Antibiotics

Antibiotic or antimicrobial resistance arises when the otherwise effective antibiotic or antimicrobials are no more effective in eradicating the pathogen. Resistance is noticed, or any bacterium is called resistant, if bacteria even survive at higher dose of permissible drugs. When the antibiotic is not able to kill the pathogen then the term is equivalent to drug tolerance or drug failure. When the organism becomes resistant to more than one type of antibiotic then the organism is referred to as multidrug resistant. Bacteria acquire these features with time to resist an earlier effective drug. Bacteria evolved number of mechanisms of resistance. Particularly, for *β*-lactams, thus far four major ways have been known to us that are used by host bacteria to avoid the bactericidal effects of *β*-lactams.

(I) *β*-lactamase production that breaks the *β*-lactam ring and makes the antibiotic inactive before it reaches the PBP target. This mode of mechanism is very common in* Enterobacteriaceae* such as* Escherichia coli* and* Klebsiella* [52]. We will be mainly focusing on ESBL resistance in the next sections.

(II) Expressing altered and mutated PBPs, this mechanism is responsible for resistance to penicillin in* Pneumococci* and methicillin resistance in* Staphylococci* [53].

(III) Absence or reduced expression of outer membrane proteins (OMPs) in Gram-negative microbes [54].

(IV) Overexpression of efflux pumps, a system that ejects the antibiotics out of the cell with energy expenditure, thereby decreasing intracellular concentrations of the antibiotic. Essentially, this active efflux system is comprised of a complex of specialized proteins that form a bridge between the cytoplasmic membrane and the outer membrane. A carrier protein in the cytoplasmic membrane, capable of capturing molecules located in the membrane or the cytoplasm, is linked to an “accessory protein,” connected in turn with an outer membrane protein channel. Although the efflux of antimicrobials is the most common mode of acquired resistance to tetracyclines, it has been involved in developing resistance to other drugs including *β*-lactams [55].

## 3. The **β**-Lactamases

These enzymes are produced by microorganisms and are able to break *β*-lactam molecules rendering them inactive and thus singularly or in part enable *β*-lactam resistance. So far, more than 500 *β*-lactamases have been reported to date (https://www.lahey.org/studies/) produced by diverse bacteria. Beta-lactamases are thought to be the most common resistance mechanism that contributes to widespread resistance among Gram-negative microbes [56]. In Gram-negative microbes, the *β*-lactamase mediated resistance is either plasmid mediated or expressed chromosomally. Nevertheless, the spread of *β*-lactamases is frequently linked with plasmid mediated ESBLs, specifically the CTX-M family [57]. In Gram-negative organisms, chromosomally located inducible expression of *β*-lactamases is also common, while plasmid mediated enzymes are generally expressed constitutively.

Generally, two classification systems for these enzymes are currently in use. The first is Ambler molecular classification or molecular classification (Table 1), which is based on the conserved motifs and protein sequence that further categorizes these enzymes into four such as classes A, B, C, and D enzymes. These enzymes utilize serine for *β*-lactam hydrolysis and class B metalloenzymes that require divalent zinc ions (metal ion) for substrate hydrolysis. The second categorization is named as Bush, Jacoby, and Medeiros functional classification, which groups different *β*-lactamases according to their substrate and inhibitor profiles. This method correlates the beta-lactamases with phenotypes in clinical isolates. The latest updated functional classification is comprised of three groups: group 1 (class C), cephalosporinases; group 2 (classes A and D), broad spectrum, inhibitor-resistant, extended spectrum *β*-lactamases, and serine carbapenemases; and group 3 (class B), metallo-*β*-lactamases [56, 58] (Table 1). Group 1 enzymes are usually encoded in the chromosome and are usually resistant to inhibition by clavulanic acid. AmpC expression is low, but when accumulated in large amount in the host cells it can offer resistance against carbapenems, mainly against ertapenem [46] (Table 2). AmpC has been extensively reviewed by other authors [59–63] and will not be reproduced here. The traditional plasmid mediated CMY (blaCMY-1-136), ACT, DHA, FOX, and so on are now outnumbered by the common plasmid mediated subgroup 2be ESBLs. Subgroup 2a penicillinases, although a small group and with limited spectrum, are predominant *β*-lactamases produced by Gram-positive cocci such as staphylococci. Notably, subgroup 2b containing TEM-1, TEM-2, and SHV-1 enzymes efficiently hydrolyzes penicillins and early cephalosporins (cephaloridine) and is strongly inhibited by the clavulanic acid and tazobactams. Furthermore, group 2be enzyme exhibits extended spectrum activity that hydrolyzes one or more oxyimino-*β*-lactamases (cefotaxime, ceftazidime, and aztreonam), derived of amino acid substitution in TEM-1, TEM-2, and SHV-1 [64].

## 4. Extended Spectrum **β**-Lactamases (ESBLs)

Among the *β*-lactamases, ESBLs are worthy of the attention of the scientific community over the last decades. Generally, ESBLs are plasmid born and are known for their ability to hydrolyze oxyimino-cephalosporin (3rd- and 4th-generation cephalosporins) and monobactams but not cephamycin such as cefoxitin and carbapenems comprising meropenem, imipenem, ertapenem, and doripenem. Furthermore, these are generally susceptible to *β*-lactamase inhibitors such as clavulanic acid, sulbactam, and tazobactam. Classically, ESBLs are defined as enzymes originally derived or evolved from a narrow spectrum parent ESBL-enzyme and thus gained the ability to inactivate the broad spectrum cephalosporins, penicillins, and aztreonam, but not the cephamycins (cefoxitin) or carbapenems by hydrolytic activity and are inhibited by *β*-lactamase inhibitors, that is, clavulanic acid. The older and classical definition of ESBL includes derivatives from TEM-1, TEM-2, or SHV. Most recent definition divides ESBL into three main groups.

(i) ESBL_A_ (class A ESBLs) comprises the most frequently found ESBL and the CTX-M, as well as SHV and TEM enzymes. These enzymes are mainly horizontally transferable and can be inactivated or inhibited by clavulanic acid.

(ii) ESBL_M_ (miscellaneous ESBLs) are sectioned into ESBL_M-C_ (class C, plasmid mediated AmpC) and ESBL_M-D_ (class D). Acquired AmpC are the most frequently found ESBL in this class.

(iii) ESBL_CARBA_ (ESBLs which degrade carbapenems) are divided into ESBL_CARBA-A_, ESBL_CARBA-B_, and ESBL_CARBA-D_ [65]. ESBLs are often found carried on large plasmids in addition to other resistance genes that confer resistance to antimicrobials such as aminoglycosides and sulphonamides [56].

## 5. Types of ESBLs

Among the many ESBLs described in a variety of pathogens, CTX-M, TEM, and SHV types proved to be the most successful in terms of promiscuity and dissemination across various epidemiological niches. It is thought that two main evolution strategies have been adopted by various Gram-negative bacteria such* E. coli* and* K. pneumoniae*: (i) the assortment of mutants with extended substrate specificity from the already prevalent TEM and SHV types of *β*-lactams; and (ii) uptake and capturing of novel broad spectrum *β*-lactamases genes from the naturally existing metagenome, coding for enzymes naturally endowed with ESBL activity. Various reviews have been published on the types of ESBL *β*-lactamases [65, 66]; however, they are evolving so rapidly that regular review of the accumulated knowledge on the structure and functional diversity of ESBL is necessary to update the readers. The pace at which new types of ESBLs are reported can be understood from a report published in 2005 describing 138 TEM types, 62 SHV types, and 39 CTX-M types on the 25th day of January, while currently, more than 150 of each of these variants have been described (see below).

### 5.1. CTX-M

Although a bit recently discovered, CTX-M enzymes are the most increasingly reported types of enzymes associated with resistance. CTX-M enzymes are plasmid-based encoded cefotaximases that constitute the fast growing family of ESBLs [65]. CTX-Ms are named after their extended activity against cefotaxime compared to ceftazidime and the origin of its first isolation (Munich, Germany) [67]. Among other ESBLs, CTX-M enzymes have been proven to be the most efficacious in terms of promiscuity and its predominance abundance in diverse epidemiological settings, where they have largely replaced and outnumbered other ESBL types such as TEM [68]. Thus far, 172 CTX-M (access on 14 January 2018) variants have been reported to date (http://www.lahey.org/Studies/other.asp#table1) with distinct amino acid sequence and functional characteristics. CTX-M expression is quite often associated with coresistance along with expression of other resistance elements critically reducing response to treatment. Unlike the TEM and SHV ESBLs (see below), CTX-M type enzymes did not arise as a result of alterations of existing enzymes; they were acquired de novo by lateral gene transfer from* Kluyvera* sp. [68]. A phylogenetic tree can be drawn based on the amino acid sequence to determine the relatedness among the members of CTX-M *β*-lactamases. CTX-M has been divided into six sublineages or groups (CTX-M-1, CTX-M-2, CTX-M-8, CTX-M-9, CTX-M-25, and KLUC, entitled after the first member of the group that was described). Members within a group have >94% amino acid relatedness and ≤90% relatedness across the groups. Additionally, there are about four CTX-M variants that exhibit a hybrid structure, namely, CTX-M-45 (formerly Toho-2), which is a hybrid of CTX-M-14 with a protein of unknown origin, and CTX-M-64, CTX-M-123, and CTX-M-132 that are hybrids of CTX-M-15 with different segment CTX-M-14 [65]. While the main variants of CTX-Ms are biologically different, CTX-M-15 and CTX-M-14 are the most common variants detected globally in important microbes, followed by CTX-M-2, CTX-M-3, and CTX-M-1 [69]. In the early 1990s, reports from distant countries suggested the potential of spread of these enzymes and its ability to disseminate. During this time, diversification was also noticed, illustrated well by the CTX-M-3, closely related to CTX-M-1 differing in four amino acid positions (V77, D114, S140A, and N288D). In this context, CTX-M-10 was reported in the Mediterranean areas [70] and CTX-M-15 in New Delhi [71]. The CTX-M-10 differs in two amino acids (at positions A27A and R38Q) from CTX-M-3, while CTX-M-15 differs in a single amino acid at position (D240G); presumably, all these three might have been produced from a common ancestor.

### 5.2. TEM ESBLs

TEM are mostly encoded by Gram-negative bacteria. Almost 90% of the resistance against ampicillin in Gram-negative bacteria are due to TEM encoded genes [72]. The TEM-type ESBLs are often plasmid mediated derived from mutations in the classic TEM (TEM-1 and TEM-2) genes by single or multiple amino acid substitution around the active site.* E. coli*, isolated from a patient named Temoneira (hence, named TEM) in Athens, Greece, harboring resistance encoded by* TEM-1* was the first ever report in 1965 [73]. TEM-1 is able to hydrolyze penicillin and 1st-generation cephalosporin such as cephaloridine. TEM-2 derived from the original TEM-1 enzymes as a result of single or multiple amino acid sequence mutations. All of them have a similar hydrolytic profile but each one has different isoelectric point, and hence not considered as ESBL [65]. In 1987* Klebsiella pneumoniae* isolates spotted in France as early as 1984 were found to harbor a new plasmid mediated *β*-lactamase coined CTX-1 because of its greater activity against cefotaxime. The enzyme, now called TEM-3, differed from TEM-2 by double amino acid replacements [74]. Later on, TEM-5 and TEM-4 were discovered that were found 3 and 4 amino acid different from the parent TEM-1 [74]. TEM-12 was the 1st TEM-type ESBL detected in* Klebsiella oxytoca*, isolated in Liverpool, England, in 1982 [75]. The list is also growing for TEM as new and novel variants are being reported from almost all over the world with the number raising to 223 (14 January 2018) and can be accessed at http://www.lahey.org/Studies/temtable.asp.

### 5.3. SHV ESBLs

SHV types of enzymes are mostly found in* Klebsiella* species (especially* K. pneumoniae*) most often housed by a plasmid. However, a number of species have been shown to carry SHV-1 gene within the chromosome [65]. SHV denotes sulfhydryl variable as it was believed that the inhibition of SHV activity by chloromercuribenzoate was substrate-related and was found inconstant according to the substrate used for the assay [76]. SHV-2 was the 1st SHV-ESBL type detected in* Klebsiella ozaenae* isolated from Germany, in 1983. This enzyme originated from point mutation in SHV-1 which resulted in substitution of glycine by serine at the 238 positions and extension of its hydrolytic substrate profile to include cefotaxime and to a minor degree ceftazidime [77]. Unlike TEM and CTX-M, SHV has few variants. So far, 193 different variants, based on the amino acid sequence composition, have been reported and available in the data base that can be accessed at https://www.lahey.org/Studies/. An online data base of THE Lac TAMASE E NGINEERING D ATABASE (http://www.laced.uni-stuttgart.de/) has so far 132 assigned SHV types with structural and functional characteristics. Moreover, the substation of amino acid and position of substation is restricted to a limited and narrow region unlike the broad area of CTX-M.

### 5.4. OXA ESBLs

The OXA type is remarkably increasing family of ESBLs. These *β*-lactamases differ from the TEM and SHV enzymes in that they fit into molecular class D and functional group 2d exhibiting oxacillin-hydrolyzing capabilities [78]. They mainly have been reported in* Pseudomonas aeruginosa* unlike TEM and SHV which are prevalent in* Enterobacteriaceae.* Most of these enzymes are resistance to ampicillin and cephalothin with high hydrolytic activity against oxacillin and cloxacillin, but poorly inhibited by clavulanic acid and cannot degrade the newer cephalosporins so they are not viewed as ESBLs. However, OXA-10 destroys (weakly) cefotaxime, ceftriaxone, and aztreonam, giving most microbes reduced susceptibility to these antimicrobials. Other OXA ESBLS include OXA-11, -14, -16, -17, -19, -15, -18, -28, -31, -32, -35, and -45 [79]. Altogether, OXA type *β* lactamases is explosively increasing based on the amino acid sequence variations and so far 498 variants have been reported and arranged in the database (http://www.lahey.org/Studies/other.asp#table1).

### 5.5. Minor Extended Spectrum *β*-Lactamases

During the last ten years, class A *β*-lactamases have been designated, including SFO, BES, BEL, TLA, GES, PER, and VEB types. Some of these minor ESBL are infrequently identified or are very restricted; others are becoming locally prevalent or are progressively isolated globally.

## 6. Global Epidemiology of ESBLs

No doubt, the inception of the ESBL in clinical practices around 1980s had been seen as breakthrough against the most fatal and prevalent bugs which mainly belong to* Enterobacteriaceae*. However, this success resulted in massive use of cephalosporins that provide a selective pressure for the emergence of new variants of ESBLs. Major pathogens belonging to* E. coli*,* Salmonella*,* Klebsiella*, and* Proteus* have exploited two main schemes of ESBL evolution: (i) the selection of enzyme mutants with the ability of expanded substrate from already abundantly available plasmid mediated TEM and SHV type *β*-lactamases and (ii) the ability to acquire and integrate new *β*-lactamase genetic material from the ecological metagenome, coding enzymes that are naturally capable with ESBL action. These features made ESBL the most efficacious in spreading in the environment and clinical settings. On top of that the difficulty in treating bugs producing ESBL, mainly due to the ability of these enzymes to inactivate extended spectrum of newly synthesized *β* lactam drugs, made them hard to treat. Furthermore, at the wider level of environmental scale, the occurrence of ESBL is getting hard to treat due to number of causes such as difficulty in detection and inconsistency in reporting [69, 73]. Number of factors such as the geographical areas like country, hospitals, community, and so on, host and various reservoirs, and the ability of the mobile resistant elements to spread within environment, water, and wild animals and from food animals to human make the epidemiology of ESBL quite complicated. The first ESBL report was identified in Germany in 1983, but very soon France and the United States witnessed the cases of ESBL with severe life and economic consequences [72]. During the late 1990 and in the beginning of the 2000, a sharp increase in the dissemination of ESBL among various pathogens belonging to* E. coli* and* Klebsiella* species were reported within almost all epidemiological setting such that they become a major threat in nosocomial infections. Throughout the 1990s, they were reported primarily as members of the TEM- and SHV-beta-lactamase families in Gram-negative bacteria such as* K. pneumoniae* resulting in infection epidemics in the hospital settings. Presently, they are mostly recognized in* E. coli* that causes community acquired infections and with growing frequency contain CTX-M enzymes. This is more worrisome of the ESBL resistance features of switching over between pathogens and emergence of the novel fellow of the ESBL family.

The prevalence of ESBL producing bacteria differs a lot in Europe and there are reports on the overall levels of invasive infections caused by drug insensitive microbes in the European nations accessible annually at https://ecdc.europa.eu/. Spreading of specific clones or clonal groups and epidemic plasmids in public and hospital settings has been the main cause for the increase in most of the prevalent ESBLs belonging to the TEM (TEM-24, TEM-4, and TEM-52), SHV (SHV-5, SHV-12), and CTX-M (CTX-M-9, CTX-M-3, CTX-M-14, or CTX-M-15) families in EU. Coselection with other resistances, especially to fluoroquinolones, aminoglycosides, and sulfonamides, worsened the situation. The appearance of widespread clones hiding several beta-lactamases concurrently (ESBLs, metallo-beta-lactamases, or cephamycinases) and of new mechanisms of resistance to fluoroquinolones and aminoglycosides emphasizes regulated and concise future surveillance studies. The increase in ESBL reports was noticed even in countries with narrow use of antibiotics. In Sweden for example, since 2007 to 2011 a 100% increase in ESBL epidemics has been reported [80]. Furthermore, there have been reports of ESBL clonal outbreaks at various hospitals. In Sweden, an outbreak of CTX-M-15 carrying* K. pneumoniae* and* E. coli* was reported as a result of nosocomial infections causing huge mortalities in neonates [74]. Interestingly, over the decades, the pattern of epidemiology of ESBL is switching and changing between the strains and types of the ESBL expressing, consequently hitting different ecological niches. In the late 90s, most of the ESBL were of SHV and TEM origin and were mainly associated with nosocomial infections in the ICUs. Furthermore, the prevalence of ESBL was observed more prevalent in* E. coli* and* K. pneumonia* [81].

Alongside this, new strains of ESBL carrying with novel types of enzymes of SHV and TEM types are emerging in the Europe indicated by recent reports. Recently, from Spain, isolates of Salmonella were reported carrying TEM-52 [75], while reports indicated that CTX-m-9 are common in Spain [82]. Soon after this, a similar resistance feature carrying TEM-52 enzymes* E. coli* was reported from the United Kingdom broiler chicken farms indicating the quick and successful spread of the emerging resistance features around the globe [76]. In the Eastern Europe, however, CTX-M-3 is more common [81]. From Italy in 2011, novel SHV-12 and* E. coli* and* K. pneumoniae* have been described from different sources [77]. During the last few years, this situation changed dramatically, and most of the ESBL producers are* E. coli* and CTX-M type beta lactamase producers. Of note, unlike SHV and TEM, epidemiology of CTX-M is complex because of its association with spread of specific different mobile elements rather than the clonal expansion of the bacteria itself [83]. In addition, most of the epidemics associated with ESBL CTX-M types were reported from community acquired infections. The complexity of the epidemiology of the ESBL and particularly of CTX-M is counted because of the fecal widespread route. The overall prevalence is rapidly increasing among* E. coli* and* Klebsiella* isolates in Europe. The magnitudes of such isolates that may express ESBLs are 28% in Bulgaria, 16% in Cyprus, and 12% in Romania (https://www.rivm.nl/earss/result/). Also the MYSTIC (meropenem yearly susceptibility information collection system) indicated a drastic increase from 2.1% in the late 1990s to 10.5% until 2008. However, situation in the United States is unlike that of Europe where drug resistant* E. coli* are less prevailing than in Europe, while the MYSTIC data indicted a decline of ESBL* E. coli* producers from 5% to 2% during the last few years.

Data regarding ESBL producing* Enterobacteriaceae* in the Middle East countries are worrisome, and this region seems to be a part of the major epicentres of the global ESBL pandemics. A study conducted on samples taken from the urinary tract infections of patients indicated 60.9% of ESBL producing* E. coli* indicating an extremely high prevalence of resistant* E. coli* pathogen. Genotyping of the strains showed that all these isolates were carrying CTX-M (CTX-M-14, CTX-M-15, and CTX-M-27) and TEM type of genes [84]. Later on, in 2008, a random survey conducted on patients indicated 27% prevalence of* K. pneumonia* carrying ESBL genes of SHV-12 and TEM-1 [85], while* E. coli* have been reported with a frequency range of 10% that were producing ESBL [86].

The situation in Asia and particularly in South Asia is quite worrisome. It is more probable that, specifically in India and China, where high incidence of ESBL has been reported in early and late 1990s, CTX-M type producing bacteria have been expanded and took over other types as like in other parts of the world. In the early 1990s, reports indicate that SHV-5 and SHV-12 were more dominant in Korea and Japan [87, 88]; recent studies however indicate that CTX-M is the most dominant genotype of ESBL producers in Asia including China with exception to Japan where CTX-M-2 type has been widely disseminated [89–91]. The rate of ESBL expressing* E. coli* has been described as high as up to 68% in India [92], up to 52% in Pakistan [93], and 30% in China [94]. Our recently unpublished preliminary results based on data collected from poultry and livestock animals and their environment indicate a similar higher range of ESBL producers. More worrisomely, situation in Pakistan is quite alarming [93, 95], in part, due to missing data regarding concise surveillance and estimation of the spread of ESBL producers and due to current trends of overuse of antibiotics in hospital settings, community, livestock and poultry sectors, and agriculture.

## 7. Genetic Factors Contributed to Successful Dissemination of ESBLs

Factors associated with genetics contributing to current highly penetrating global dissemination of ESBL are poorly understood. However, molecular epidemiology of ESBL indicated a strong association of ESBL spread with conjugative plasmids and successful clones [96]. Mobile genetic elements with the ability to jump between different loci of the same chromosome or from chromosome to plasmid or vice versa have been shown implicated in the spread of resistance elements. Therefore, it is crucial to understand the molecular features of the plasmids and associated genetic elements that help resistance elements to transfer between species. In addition to circular plasmid with high efficiency of transformation, transposons are DNA fragments able to move from one place to another in the bacterial nucleic acids through transposition and can be inserted nonspecifically at any place in the bacterial nucleic acids. There are numerous different kinds of transposons, but normally they contain a transposase promoting transposition, overturned repeats in the ends, and short direct repeats of target nucleic acids bracketing the transposons. Conjugative transposons are one more type holding genes for conjugative transfer from one cell to another. Though it rarely happens and is highly regulated, transposition is one of the genetic factors that contribute to spread of resistance elements. The most important element that has been shown highly associated with successful transmission of resistance elements is integrons [90]. These are genetic elements incorporated in transposons found on sets of plasmids and in the bacterial chromosome. These gene capturing systems are progressed from site-specific recombination mechanisms, and a general integrin encodes a DNA integrase gene* (int)* and a neighboring recombination site* (att1)* [97]. Generally, within the variable region, multiple resistance associated genes could be integrated in the form of gene cassettes that are basically incorporated in the attachment site (attI) of the integron which can have many cassettes at once. Different integrons classes have been described, and classes 1, 2, and 3 have been associated with antibiotic resistance. Most often, they encode for multiple resistances and thus associated with coresistance. Importantly, integrons by itself are not able to jump, but their gene cassettes could be mobilized as they are often found as part of the transposons or plasmid and thus could be integrated into secondary sites, thereby conferring new resistance phenotype. Therefore, plasmid replicon typing is quite useful technique to analyse the strength of the ESBL to be associated with other coresistance and types of integrons that have potential of promiscuity and no specific integration and transmission. It has been suggested that the association of IncFII plasmid encoding ESBL type CTX-M-15 in the well-adapted strain of* E. coli* ST131-O25:H11 is linked to its successful widespread global dissemination [96, 98].

In addition to those described elements, other genetic elements such as toxin-antitoxin systems have been pointed that bacteria most probably would use this system to maintain the resistance elements [99]. A very recent phenomenon of multiple addiction systems was reported in plasmids bearing bla CTX-M types dependent on the multiple addiction system for plasmid maintenance and probably transmission [100]. An addiction system or a toxin-antitoxin system helps sustain plasmids in bacteria while replicating in the host by eliminating/killing plasmid-free cells resulting from segregation or replication defects. In short, this is vital to understand the genetic structural and functional elements that contribute to promote maintain and spread of resistance elements either directly or indirectly.

The insertion sequences (IS) on the one hand can cut and paste ESBL encoding genes between the plasmids and, on the other hands, can enhance the expression of these enzymes. The most prevalent IS reported so far and associated with dissemination of resistance elements are* ISEcp1*,* ISCR1, *and* IS26* [90].

## 8. Factors Affecting the Spread and Considerations

To simplify the resistance phenomenon a bit, we can keep working and concentrate on two main factors, the antibiotics themselves that provide selective pressure to dominate which have the second important element/factor: the genes. If, for example, either the antibiotic or the resistance genes did not exist, we would not have the phenomenon of resistance after all. In fact, referring to the clinical cases, finding of resistance to new drugs by a pathogen is not unexpected, as antibiotics and other organic molecules resembling antibiotics are always found in the environment. Bacteria, after their many millennia of existence, might have confronted them at some point and that would have affected their growth; to survive, bacteria would have acquired resistance to these molecules. However, the emergence of these traits in the clinical isolates or in the hospital setting is what actually warns the clinicians about the use of certain drugs. In this respect, discovery of the new resistance elements in bacteria, for example, in the commensal, foretells future complications in the pathogens in clinical isolates of that hospital, community.

In the United States alone, an estimated 9.45 × 10^6^ kg of antibiotics is used annually; half of this is provided to people for use during sickness and the rest for agricultural use [78]. These drugs in hospitals are commonly delivered parenterally, while in the community they are delivered through oral prescription. About 7 × 10^6^ kg of antibiotics, mainly penicillin and tetracycline, are routinely used annually in animals as growth promoters [78]. On top of that an estimated 4.5 × 10^4^ kg of antibiotics, chiefly tetracycline and streptomycin, are sprayed over the fruits and crops annually. Most of these would certainly reside in environment as residues for a bit and bacteria would confront them at a point. A pool of genes resistant to these antibiotics would certainly arise in the environment in response to selective pressure provided by the existing residues of these antibiotics in the environment, body and food. Above is the amount used in a country where antibiotics are narrowly and strictly prescribed. We do not have an existing data to estimate the amount of antibiotics that are used in Pakistan. But based on the prevailing practices about how antibiotics are provided in country, it is nevertheless hard to estimate that we are currently using higher amount of antibiotics in clinical, agriculture, and food animals. Antibiotics in Pakistan are available in the pharmacy stores, and even vendors are selling them out on the streets. This means that drug distribution provides the worst scenario for emergence of antibiotic resistance, the possibility of too little options of drugs for treatment of severe patients and provision of drugs when they are not needed. This blind use of antibiotics is thus encouraging new elements of resistance to emerge against antibiotics. In this way, antibiotics are excreted in the environment, water, crops, and so on, where the antibiotics keep exerting selective pressure resulting in “posttherapy” environmental selection phase of the antibiotic [79]. During such phase, the antibiotic concentration would be less than the therapeutic concentration, which is ideal for selecting resistance. Thus, considering this scenario, it may not be the use of persistence high concentration of antibiotics in clinics for treatment (treatment period), rather the slow and persistence release of low amount of antibiotics in the environment (posttherapy period) that provide ample amount of time for bacteria to develop resistance. Altogether, we should really change our mentality and course of action against the use of antibiotics. First, we should emphasize implementing the shorter course of antibiotics. Secondly, cycling of the antibiotics should be considered if new antibiotics are available in the market. Thirdly, we should alongside invest in synthesizing or discovering new antibiotics such as improvement of the current tetracycline drugs; understanding the mechanism of resistance to currently available tetracycline drugs would help improve the efficacy. A chemical free, nonclassical approach to retreat the resistance problem would be the recovery of the vulnerable strain. We need to encourage the growth of the susceptible strain to take over the resistance one. One of the approaches would be to reintroduce the susceptible flora in the form of probiotic. As we understand more and more the chain of producing resistance elements that provide impetus to the rise of antibiotic resistance in human, animal, food, and environment, we need to block those chains and encourage the forward lane for improving the susceptible strains. Also instead of attacking and jumping from one factor to another, sticking to one of the responsible factors and eliminating it eventually would help us to eliminate resistance bacteria. We need to bring “peace” instead of attacking and conquering the bacteria. Commensals bacteria are our allies and we need to encourage them and to take over the resistance one. Thus, a time will arrive that once we have been sick of bacteria, they will be susceptible to antibiotics and would be easy to eliminate.

## 9. Conclusions

Resistance to antibiotics may not be surprising, but the current rise in resistance against the vital antibiotics and its acquisition by commensal bacteria is quite worrisome. Elements that enable bacteria to neutralize the toxic effects of an otherwise effective drug are attributed to the genetic elements that are either encoded in the chromosome and thus vertically propagated or acquired horizontally from the environment. The latter case asks for more attention as it is usually associated with transferable elements and coresistance for other important drugs. At the moment, most of the resistance elements that can inactivate extended spectrum *β*-lactam drugs are encoded in the transferable elements such as plasmids with the ability of its promiscuity and chances of spread in the gut, environment, and food animals that is acquired by similar or different bacteria even turning commensals into pathogens. To better prepare before any epidemics of the resistant bacteria collapse the current health standards and achievements, we must understand the structural and genetic background of these resistance elements. We should not underestimate the impact of antibiotic prescription at any level on the spreading and determination of resistant microbes. Intending to use these vital compounds as rational as possible, we need to further broaden our concept about how resistance arises and all the factors effecting its spreading. We need to optimize the antibiotic treatment by enhancing diagnostic tool to provide faster and accurate diagnosis for the use or the intended antibiotic to avoid empirical treatments. Much of further research and investment is needed in all these aspects. Strains and plasmid interaction and propagation ask for more attention to further enhance our understanding. Much more is needed to know the persistence strains and means of emergence of new strains both in healthy and in sick population. This way, we could collect information that help us handle patients infected with ESBL bacteria in hospitals and for how long persistence and dissemination would be expected from a patient in different environments under various conditions. This could also help us know how to adjust antimicrobial treatment to be as effective as possible and give little chances to resistant bacteria to grow under the presence of selective pressure. New antibiotics discovery, new treatment options, and optimization of the use of already available antibiotics would buy us time to address this important public health issue.

## Figures and Tables

**Table 1:** Various classification schemes and representatives of extended spectrum beta-lactamase enzymes.

Ambler (molecular) class	Bush & Jacoby group (2009)	Substrate/target	Inhibition profile		Member examples
			Clavulanic acid	Tazobactam	
A	2a	Penicillins	Yes	No	PC-1
	2b	Penicillins, some of the 1st-generation cephalosporin	Yes	No	TEM-1, TEM-2, SHV-1
	2be	Extended spectrum cephalosporin, monobactam	Yes	No	TEM-3, SHV-2, CTX-M-15, PET-1, VEB-1
	2br	Penicillins	No	No	TEM-30, SHV-10
	2ber	Extended spectrum cephalosporin, monobactam	No	No	TEM-50
	2c	Carbenicillin	Yes	No	PSE-1, CARB-3
	2ce	Carbenicillin, cefepime	Yes	No	RTG-4
	2e	Extended spectrum beta-lactams	Yes	No	CepA
	2f		Changing	No	KPC-2, 1M1-1, SME-1

B	3a	Carbapenems	No	Yes	IMP-1, VIM-1, CcrA, IND-1, NDM-1
	3b	Carbapenems	No	Yes	CphA, Sfh-1

C	1	Cephalosporins	No	No	AmpC, P99, ACT-1, CMY-2, FOX-1, MIR-1
	1e	Cephalosporins	No	Yes	GC1, CMY-37

D	2d	Cloxacillin	Changeable	No	OXA-1, OXA-10,
	2de	Extended spectrum cephalosporin	Changeable	No	OXA-11, OXA-15
	2df	Carbapenems	Variable	No	OXA-23, OXA-48

**Table 2:** List of bacteria expressing identified AmpC *β*-lactamases.

Origin (bacteria)	Designated enzyme/gene name	GenBank/protein accession number	Reference(s)
Chromosomal Ampc			
*Aeromonas caviae*	*AmpC*	AAM46773	[15]
*Aeromonas hydrophila*	*AmpH and CepH*	YP_857635	[16]
*Aeromonas jandaei*	AsbB1 and AsbA1	AAA83416	[17]
*Aeromonas salmonicida*	*AmpC*	ABO89301	[18]
*Aeromonas veronii* bv. sobria	AmpS. CepS	CAA56561	[19]
*Buttiauxella agrestis*	BUT-1	AAN17791	[20]
*Citrobacter braakii*	*AmpC*	AAM11668	[21]
*Citrobacter freundii*	*AmpC*	AAM93471	[22]
*Citrobacter murliniae*	*AmpC*	AAM11664	[21, 23]
*Citrobacter youngae*	*AmpC*	CAD32304	[23]
*Citrobacter werkmanii*	*AmpC*	AAM11670	[21]
*Edwardsiella tarda*	blaC	ABO48510	[24]
*Enterobacter aerogenes*	OCH-1	AAO16528	[25]
*Enterobacter asburiae*	*AmpC*	CAC85157	[26]
*Enterobacter cancerogenus*	*AmpC*	AAM11666	[21]
*Enterobacter cloacae*	*AmpC*	P05364	[27]
*Enterobacter dissolvens*	*bla*_ACT-1_	CAC85359	[26]
*Enterobacter hormaechei*	*bla*_ACT-1_	CAC85357	[26]
*Escherichia fergusonii*	*AmpC*	AAM11671	[21]
*Escherichia coli*	*AmpC*	NP_418574	[28]
*Hafnia alvei*	*AmpC*	AAF86691	[29]
*Morganella morganii*	*AmpC*	AAC68582	[30]
*Providencia stuartii*	Type I *β*-lactamase	CAA76739	[31]
*Serratia marcescens*	Class C beta-lactamase	AAK64454	[32]
*Shigella boydii*	*AmpC*	YP_410551	[33]
*Yersinia enterocolitica*	*AmpC*	YP_001006653	[34]
*Pseudomonas aeruginosa*	*AmpC*	NP_252799	[35]
*Pseudomonas fluorescens*	Class C beta-lactamase	YP_349452	[36]
*Psychrobacter immobilis*	Class C beta-lactamase	CAA58569	[37]
*Chromobacterium violaceum*		NP_900980	[38]
Plasmid mediated AmpC			
*K. pneumoniae *	*CMY1*	P71420	[39, 40]
*K. pneumoniae*	CMY-2	Q48434	[41]
*K. pneumoniae*	MIR-1	M37839.	[42]
*K. pneumoniae*	MOX-1	Q51578-1	[43]
*K. pneumoniae*	LAT-1	Q48443	[44]
*S. enteritidis*	DHA-1	O54216	[45]
*K. pneumoniae*	ACT-1	D2KFG4	[46]
*K. pneumoniae*	ACC-1	B0RZ87	[47, 48]
*E. coli*	CFE-1	Q83ZC8	[49]
